# Molecular characterization of measles virus strains circulating in Cameroon during the 2013-2016 epidemics

**DOI:** 10.1371/journal.pone.0222428

**Published:** 2019-09-25

**Authors:** Franck-Martin Obam Mekanda, Chavely Gwladys Monamele, Frédy Brice Simo Nemg, Gilde Martial Yonga, Diane Ouapi, Véronique Penlap Beng, Christophe Batéjat, Valérie Caro, Jean-Claude Manuguerra, Maurice Demanou

**Affiliations:** 1 WHO National Measles Reference Laboratory, Department of Virology, Centre Pasteur of Cameroon (CPC), Yaoundé, Centre, Cameroon; 2 Faculty of Science, University of Yaoundé 1, Yaoundé, Centre, Cameroon; 3 Cellule d’Intervention Biologique d’Urgence (CIBU), Unité de Recherche et d’Expertise ‘Environnement et Risques Infectieux’ (ERI), Institut Pasteur, Paris, France; university of campus biomedico, ITALY

## Abstract

The first genotyping data on measles virus (MeV) strains in Cameroon dates from 1994, while other studies were realized in 2001 and 2011 with the establishment of MeV virological surveillance. However, the genetic data of MeV strains circulating in Cameroon remains fragmented and concentrated in certain regions, hence the need for an update. The objective of this study was to have recent data on MeV genotypes circulating in Cameroon. Ninety throat swabs collected during recent measles outbreaks were analyzed by MeV genotyping RT-PCR using the nucleoprotein gene N. The resulting sequences were analyzed on the basis of 450 nucleotides with MEGA 7 software. Overall genome analysis was performed on 40/90 sequences. The strains were from all ten regions and all belonged to cluster 1 of genotype B3. The genotype B3 has been circulating in Cameroon for long periods of time; efforts must be made in immunization for its elimination.

## Introduction

Measles is a highly contagious and potentially fatal viral infection of the child and young adult caused by measles virus (MeV): a 15.9 kb enveloped, non-segmented, negative-stranded RNA virus belonging to the *Paramyxoviridae* family and the *Morbillivirus* genus [1]. This virus is transmitted from human to human via sputum and droplets from the respiratory tract [2].

Clinically a person is declared a suspect case of measles when he has a generalized maculopapular rash and fever, with cough and/or coryza, and/or conjunctivitis [3]. Once the disease progresses, the immunosuppression caused by measles virus (MeV) exposes the patient to bacterial super infections causing otitis media, pneumonia, or gastrointestinal infections. The main cause of measles-related morbidity and mortality is due to association of these symptoms with other factors such as malnutrition and vitamin A deficiency [4].

Before the introduction of measles immunization in the 1960s, almost everyone contracted measles usually during childhood with an estimated annual incidence of 130 million cases and an early mortality of 2.5 million each year. Since the introduction of the vaccine, the incidence of measles as well as the associated early mortality rate decreased. From 2000 to 2016, a 84% drop in mortality was noted from about 550,100 to 89,780 deaths, global annual reported incidence decreased by about 87% from 145 to 19 cases per million persons, estimates of the first dose of measles-containing vaccine (MCV1) coverage increased globally from 72% to 85% and the number of countries providing second dose of measles-containing vaccine (MCV2) nationally through routine services increased from 98 (51%) to 164 (85%) in which 24 African countries [5]. Despite this progress, epidemics have continued in some regions. In 2016, of the 89,780 deaths attributed to measles, 85% occurred in Africa and Asia [5].

To date, some countries where measles has been declared eliminated have confirmed new cases in recent years, such as, South Korea with 107 new cases, of which 04 imported in 2013 [6]. The WHO European Region which was already close to eliminating measles confirmed a total of 21,315 cases between 2016 and 2017 with 35 deaths [7]. Measles remains a global public health problem hence the need to strengthen immunization, surveillance and health monitoring.

The MeV is monotypic, but the genetic variability of the genes encoding the viral hemagglutinin (H) and the nucleoprotein (N) makes it possible to be genetically characterized in 8 clades (A-H) and 24 genotypes (A, B1-B3, C1- C2, D1-D11, E, F, G1-G3, H1-H2) [8]. Genotype B3 endemic in several African countries can be further divided into 3 clusters (B3.1, B3.2, and B3.3) [9].

As part of strategic plan for measles control and elimination, the World Health Organization (WHO) has recommended that member states implement virological surveillance for circulating MeV strains [10]. Molecular characterization of strains is a key tool in the study of measles transmission pathways, evaluation of endemic measles eradication and indirect evaluation of the vaccination system in place [11,12]. When high quality surveillance system associated with absence of endemic MeV transmission exist in certain region or defined geographic area for ≥12 months of period of time, measles can be decleared eliminated in this area or region [13].

The latest genetic data on MeV strains circulating in Cameroon have been known since 1994 [14] and were updated in 2001 [15] and 2011 [16], however they remain fragmented and are relatively old. The aim of this study was to update the MeV strains circulating in Cameroon. These results will enable to establish a genetic basis of virological surveillance in Cameroon and evaluate the country’s efforts to eradicate this disease.

## Material and methods

### Ethics statement

This study was carried out as part of measles surveillance in Cameroon at Centre Pasteur of Cameroon (CPC), the National Reference Laboratory (NRL) for measles since 2011. There was thus no need for an ethical clearance. However, all participants read an information notice and gave a verbal consent before enrollment and sample collection.

### Clinical samples

Ninety (90) throat swabs collected during the 2013, 2014, 2015 and 2016 measles outbreaks were collected by the surveillance team of the Expanded Program on Immunization (EPI) and transported to the NRL while respecting the cold chain. Once in the laboratory, samples were stored at -20 °C prior to analyses.

### Laboratory methods

RNAs were extracted directly from the swabs using the QIAamp Viral RNA Mini Kit (QIAGEN, Hilden, Germany) according to the manufacturer’s instructions. RNA extracts were then analyzed by MeV genotyping RT-PCR in search of 634 nucleotides of the N-gene with forward primer MeV214 (5'-TAACAATGATGGAGAGGGTAGG-3') and reverse primer MeV216 (5'-TGGAGCTATGCCATGG GAGT-3'). The SuperScript^™^ III one-step system with PlatinumW Taq High Fidelity enzyme was used for amplification of the target gene fragment. The RT-PCR mixture was composed of 17 μl DNAse/RNAse-free water, 25 μl of 2X buffer, 1 μl of MeV214 primer, 1 μl of MeV216 primer, 1 μl of the enzyme, and 5μl of the RNA extract. RT-PCR was programmed for 40 cycles including a reverse transcription step of 30 minutes at 50°C and 15 minutes at 95°C, a denaturation step of 30 seconds at 94°C, primers hybridization of 30 second at 55°C, an initial elongation of one minute at 72°C, a final elongation of 10 minutes at 72°C and storage at 4°C. The RT-PCR products were revealed by 1% agarose gel electrophoresis after 30 minutes migration for 250 volts. All RT-PCR positive products were sequenced using the MeV214 and MeV216 primers and the resulting sequences were edited and assembled into a single consensus using the CLC Mainworkbench 5.5 software.

### Phylogenetic analyses

Phylogenetic analyses were performed on 450 nucleotides of the N gene according to WHO recommendations for measles virus strains genotyping [17]. All consensus sequences obtained from the CLC Mainworkbench 5.5 software were first aligned with the 26 reference sequences and subsequently by 11 other sequences of genotype B3 available in GenBank using the Clustal W algorithm in MEGA 7 software. The best fit-model of nucleotide substitution was selected in MEGA 7 and was found to be the Kimura two-parameter gamma model among 24 different nucleotide substitution models. Phylogenetic trees were generated with the Maximum likelihood algorithm using Kimura two-parameter distance model in MEGA 7. Evolutionary rates among sites were modeled using the Gamma distribution and the attribution of clades, genotypes and clusters was based on the clusters formed between the studied sequences and the reference sequences of known clades. All sequences obtained in this study were named according to the WHO recommendations and submitted to Measles Nucleotide Surveillance (MeaNS) and GenBank under accession number MH255950 to MH255989.

## Results

Overall, 41 of 90 samples (45.55%) were RT-PCR positive for the genotyping assay and 40 of these sequences could be used for phylogenetic analyses. Table 1 summarizes the sociodemographic data of the subjects from which the sequenced samples were obtained. All strains were collected from 2013 through 2016: 08 in 2013, 20 in 2014, 05 in 2015 and 07 for 2016. The average age of the affected subjects was 8.6 years with extremes of 7 months and 20 years, the majority of viruses were obtained from children under 10 years of age with a predominance of those aged 7 months to 5 years (29/40). The male sex was the most predominant gendre (27/40) and 08 (20%) patients had a history of vaccination against measles.

**Table 1:** Characteristics of patients in whom MeV strains were collected in Cameroon from 2013 to 2016.

Genbank number	Strain name	Patient age (months)	Sex	Patient location (Region-District)	Last immunization date
**MH255950**	MVs/Olamze.CMR/2.13/	36	M	South- Olamze	
**MH255951**	MVs/Garoua Boulai.CMR/10.13/	12	M	East-Garoua Boulai	
**MH255952**	MVs/Loum.CMR/14.13/	72	M	Littoral-Loum	
**MH255953**	MVs/Citee des Palmiers.CMR/25.13/	10	F	Littoral-Citee des Palmiers	15/05/2013
**MH255954**	MVs/Olamze.CMR/5.13/	156	M	South- Olamze	
**MH255955**	MVs/Mifi.CMR/7.13/	72	M	West-Mifi	
**MH255956**	MVs/Foumbot.CMR/7.13/	12	M	West-Foumbot	
**MH255957**	MVs/Foumbot.CMR/6.13/	144	F	West-Foumbot	
**MH255958**	MVs/Maroua Rural.CMR/8.14/	12	F	Far Norht-Maroua Rural	
**MH255959**	MVs/Yoko.CMR/9.14/	36	M	Centre-Yoko	
**MH255960**	MVs/Mbouda.CMR/10.14/2/	36	F	West-Mbouda	
**MH255961**	MVs/Mbouda.CMR/10.14/	72	F	West-Mbouda	
**MH255962**	MVs/Bibemi.CMR/10.14/	36	F	North-Bibemi	
**MH255963**	MVs/Foundong.CMR/9.14/	48	M	North West-Fundong	14/09/2010
**MH255964**	MVs/Maroua Urbain.CMR/11.14/	36	M	Far North-Maroua Urbain	
**MH255965**	MVs/Maroua Urbain/11.14/2/	60	F	Far North-Maroua Urbain	
**MH255966**	MVs/Maroua Urbain.CMR/11.14/3/	60	M	Far North-Maroua Urbain	
**MH255967**	MVs/Kumba.CMR/26.14/	7	M	South West-Kumba	
**MH255968**	MVs/Betare Oya.CMR/38.14/	60	F	East-Betare Oya	
**MH255969**	MVs/Betare Oya.CMR/38.14/2/	12	M	East-Betare Oya	
**MH255970**	MVs/Betare Oya.CMR/38.14/3/	96	F	East-Betare Oya	
**MH255971**	MVs/Kumba.CMR/43.14/	12	M	South West-Kumba	
**MH255972**	MVs/Ngaoundere Rural.CMR/46.14/	168	M	Adamawa-Ngaoundere Rural	
**MH255973**	MVs/Touboro.CMR/46.14/	240	M	North-Touboro	
**MH255974**	MVs/Bertoua.CMR/51.14/	12	M	East-Bertoua	
**MH255975**	MVs/Nwa.CMR/3.14/	8	M	North West-Nwa	
**MH255976**	MVs/Nwa.CMR/3.14/2/	60	M	North West-Nwa	01/02/2009
**MH255977**	MVs/Garoua Boulai.CMR/8.14/	24	F	East-Garoua Boulai	
**MH255978**	MVs/Guidiguis.CMR/3.15/	36	F	Far North-Guidiguis	
**MH255979**	MVs/Guidiguis.CMR/3.15/2/	84	M	Far North-Guidiguis	
**MH255980**	MVs/Biyem Assi.CMR/8.15/	10	M	Centre-Biyem Assi	
**MH255981**	MVs/Yokadouma.CMR/8.15/	36	F	East-Yokadouma	21/10/2012
**MH255982**	MVs/Kumba.CMR/18.15/	36	M	South West-Kumba	01/11/2012
**MH255983**	MVs/Lagdo.CMR/10.16/	11	M	North-Lagdo	
**MH255984**	MVs/Lagdo.CMR/12.16/	12	M	North-Lagdo	
**MH255985**	MVs/Kumba.CMR/18.16/	24	F	South West-Kumba	02/02/2015
**MH255986**	MVs/Zoetele.CMR/38.16/2/	168	M	South-Zoetele	01/02/2003
**MH255987**	MVs/Zoetele.CMR/38.16/	36	M	South-Zoetele	01/12/2013
**MH255988**	MVs/Mora.CMR/45.16/	10	M	Far North-Mora	
**MH255989**	MVs/Bertoua.CMR/7.16/	108	M	East-Bertoua	

**Fig 1:**
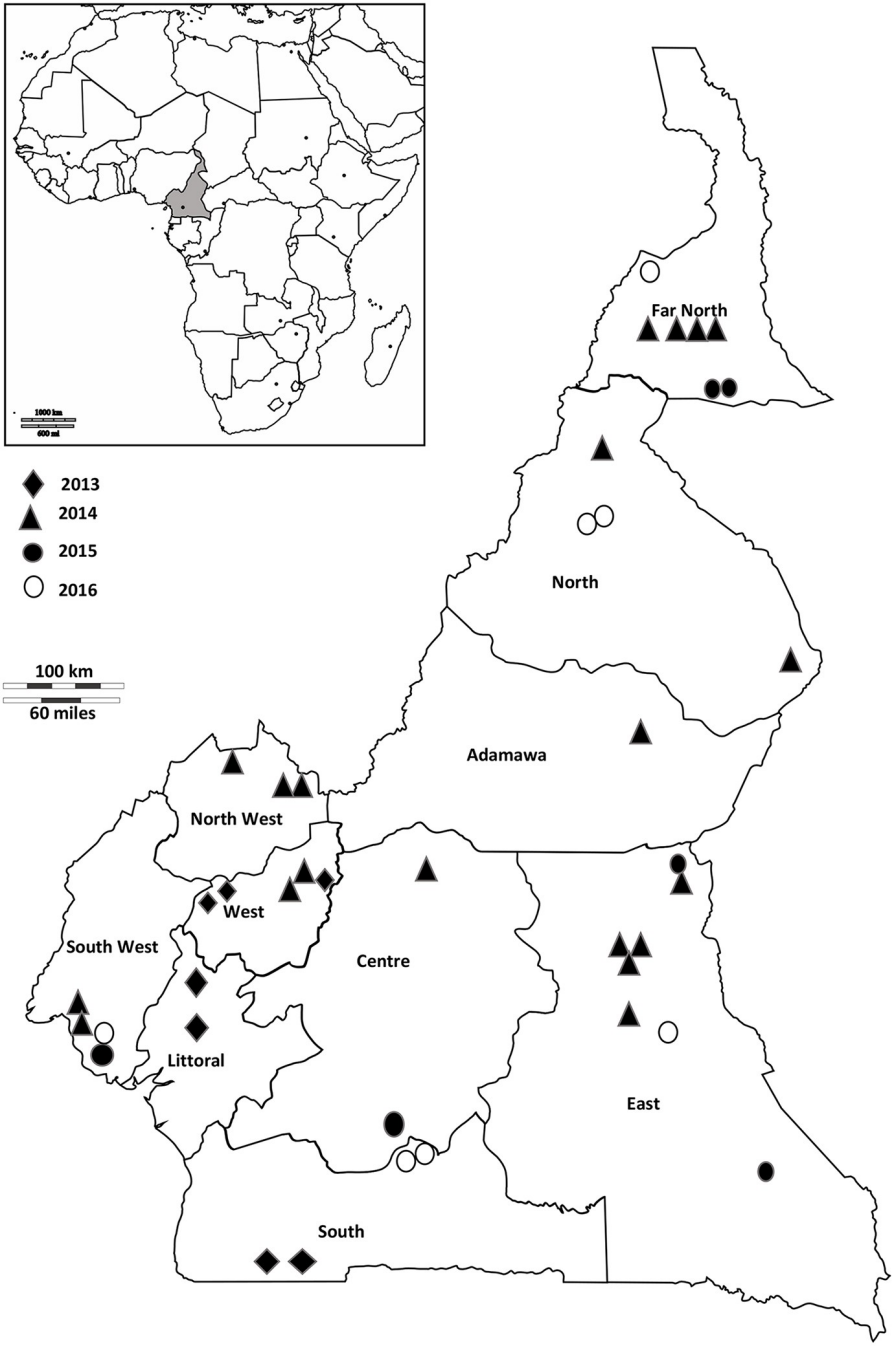
Distribution of genotype B3.1. Strains by region and year of collection diamonds (♦) represent the viruses collected in 2013, triangles (▲) for those collected in 2014, black circles (●) for viruses collected in 2015 and white circles (○) for those collected in 2016.

The sequences obtained came from all regions of Cameroon: 01 from Adamawa, 02 from the Center, 08 from the East, 07 from the Far North, 02 from the Littoral, 04 from the North, 03 from the North West, 04 from the South, 04 from the South West and 05 from the West (Fig 1, Table 1).

**Fig 2:**
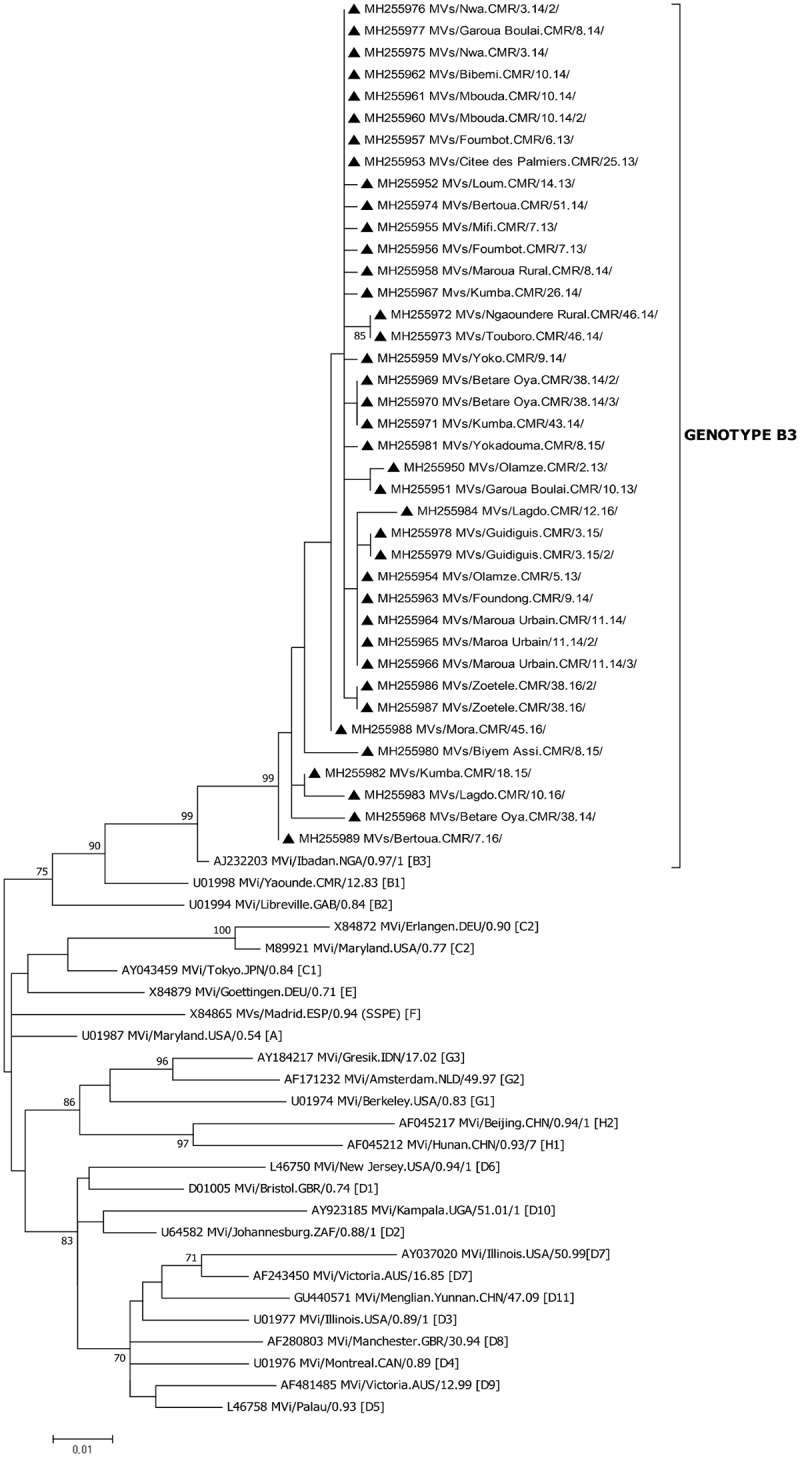
Phylogenetic tree of sequences studied with reference sequences. The studied sequences are in bold and represented by a black triangle, the reference sequences are demarked with the genotype between hooks. Accession numbers and virus names are included on the tree. Phylogenetic analysis was performed based on 450 nucleotides of the N gene in MEGA version 7.0. Evolutionary history was inferred using the Maximum Likelihood method and distances were computed using the Kimura two-parameter model. The bootstrap test was set to 1000 replicates and bootstrap values above 70% are shown next to the main tree branches.

**Fig 3:**
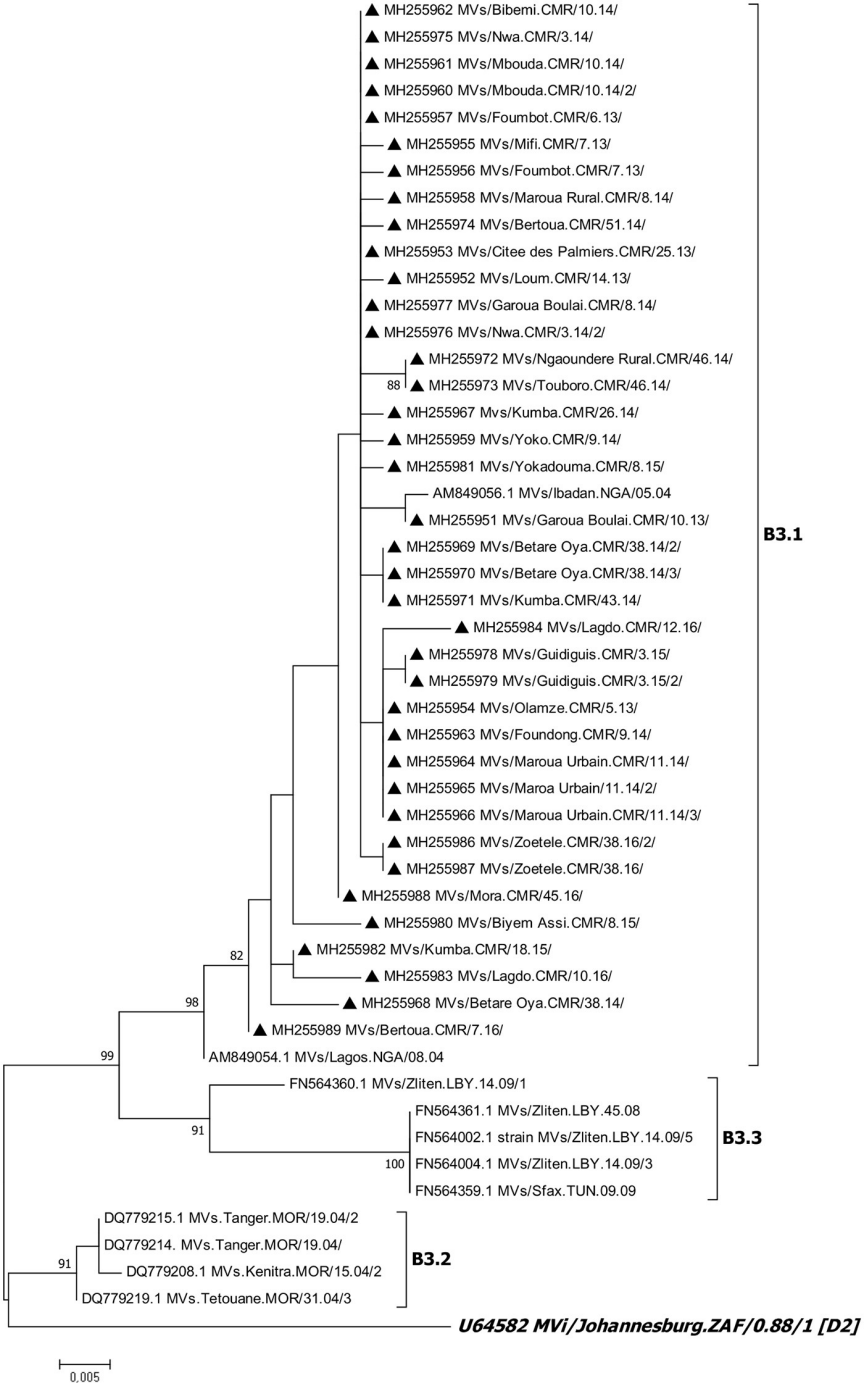
Sub genotyping phylogenetic tree. The studied sequences are represented by a black triangle. The tree was rooted with a sequence belonging to genotype D2. Accession numbers and virus names are included on the tree. Phylogenetic analysis was performed based on 450 nucleotides of the N gene in MEGA version 7.0. Evolutionary history was inferred using the Maximum Likelihood method and distances were computed using the Kimura-two parameter model. The bootstrap test was set to 1000 replicates and bootstrap values above 70% are shown next to the main tree branches.

Phylogenetic analyses showed that all sequences obtained in this study clustered with the MVi/Ibadan.NGA/0.97/1 genotype B3 sequence with a bootstrap value greater than 90% (Fig 2). A phylogenetic tree of the sub-group to which the Cameroon strains belong is shown in Fig 3. All strains belonged to cluster B3.1 with a bootstrap value greater than 90%.

## Discussion

Cameroon integrated measles surveillance in 1974 through sentinel sites in the city of Yaoundé [18]. The first genetic data on MeV date from 1994 with the identification of genotype B1 strain *Yaounde*.*CAE/12*.*83 "Y-14"* in Yaoundé [14]. Virological surveillance of MeV strains in Cameroon was effectively implemented in 2011 at the National Measles Laboratory [16]. This study is the fourth to investigate MeV strains circulating in Cameroon [14–16] and the second from the virological surveillance program set up in 2011.

The results show that the average age of the affected subjects is 8.6 years with extremes of 7 months and 20 years, suggesting that, measles remains a disease of children and young adults regardless of gender [19].

According to the phylogenetic analyses, all the sequences from Cameroon belonged to cluster 1 of genotype B3. The same cluster was detected in Cameroon in 2001 and from 2010 to 2011 [15,16]; in Nigeria and Ghana in 1998 [20]; in Sudan from 1997 to 2000 [21]; and in the Central African Republic in 2000 [22]. Cluster B3.1 strains remain endemic in Cameroon, where they have been circulating for about 17 years. This study is the first to characterize the MeV strains among Cameroonians in Adamawa, East and South regions. The East region borders the Central African Republic by Garoua Boulai and Betare Oya health districts and the South region borders Gabon and Equatorial Guinea by Olamze health district. Although there is no evidence of imported MeV strains in this study, the possibility of cross-contamination between Cameroonian populations and those bordering them is not excluded as the same genotype (B3) circulates in almost all of sub-Saharan Africa [21]. A previous study reported the circulation of this same genotype in the East region of Cameroon though it was among refugees from Central African Republic [23].

A small subset of the population (20%) from whom the samples were collected were vaccinated but no vaccine strain was detected. Keeping in mind that in Cameroon the first dose of measles-containing vaccine (MCV1) is administered at the age of 9 months [24], the patient in whom the MeV strains were detected presented rash at varying periods between 1 month and 12 years following immunization. These results reflect vaccination failures in these individuals probably due to non-development of vaccine induced protective immunity in some individuals, non-compliance to the CDC recommendations for uptake of two vaccine doses [25] or a poor conditioning of the vaccines [26].

The exclusive presence of cluster B3.1 strains in this study indicates the possible elimination of cluster B3.3 from Cameroon which was identified to circulate between 2010 and 2011 in the Far North and in the Centre regions. This suggests that, efforts made by the country for elimination of the disease are effective though should be re-enforced as the B3.1 genotype has been in circulation for over 17 years. Efforts should be put on childhood immunization since most affected children are not immunized. A second vaccine dose must as well be introduced into routine childhood immunization [13] which will allow the catch-up of failures of the first dose for a better follow-up of the recommendations for control and elimination of measles as proposed by WHO.

The study provided recent data on circulating measles viral strains throughout the country as the incidence of measles has increased worldwide. The limits of this work were the impossibility to completely sequence the studied genomes and the presence of limited samples in some years.
